# Targeting the Erk1/2 and autophagy signaling easily improved the neurobalst differentiation and cognitive function after young transient forebrain ischemia compared to old gerbils

**DOI:** 10.1038/s41420-022-00888-8

**Published:** 2022-02-26

**Authors:** Fuxing Wang, Zihao Xia, Peng Sheng, Yu Ren, Jiajia Liu, Lidong Ding, Bing Chun Yan

**Affiliations:** 1 Medical College, Institute of Translational Medicine, Department of Neurology, Affiliated Hospital of Yangzhou University, Jiangsu Key Laboratory of Integrated Traditional Chinese and Western Medicine for Prevention and Treatment of Senile Diseases, The Key Laboratory of Syndrome Differentiation and Treatment of Gastric Cancer of the State Administration of Traditional Chinese Medicine, Yangzhou University, Yangzhou, 225001 PR China; 2 Jiangsu Key Laboratory of Zoonosis, Jiangsu Co-Innovation Center for Prevention and Control of Important Animal Infectious Diseases and Zoonoses, Yangzhou, 225009 PR China; 3 Department of Neurology, Taizhou Second People’s Hospital, Taizhou, 225500 PR China

**Keywords:** Cognitive ageing, Neurological disorders

## Abstract

The hippocampal neurogenesis occurs constitutively throughout adulthood in mammalian species, but declines with age. In this study, we overtly found that the neuroblast proliferation and differentiation in the subgranular zone and the maturation into fully functional and integrated neurons in the granule-cell layer in young gerbils following cerebral ischemia/reperfusion was much more than those in old gerbils. The neurological function and cognitive and memory-function rehabilitation in the young gerbils improved faster than those in the old one. These results demonstrated that, during long term after cerebral ischemia/reperfusion, the ability of neurogenesis and recovery of nerve function in young animals were significantly higher than that in the old animals. We found that, after 14- and 28-day cerebral ischemia/reperfusion, the phosphorylation of MEK1/2, ERK1/2, p90RSK, and MSK1/2 protein levels in the hippocampus of young gerbils was significantly much higher than that of old gerbils. The levels of autophagy-related proteins, including Beclin-1, Atg3, Atg5, and LC3 in the hippocampus were effectively maintained and elevated at 28 days after cerebral ischemia/reperfusion in the young gerbils compared with those in the old gerbils. These results indicated that an increase or maintenance of the phosphorylation of ERK1/2 signal pathway and autophagy-related proteins was closely associated with the neuroblast proliferation and differentiation and the process of maturation into neurons. Further, we proved that neuroblast proliferation and differentiation in the dentate gyrus and cognitive function were significantly reversed in young cerebral ischemic gerbils by administering the ERK inhibitor (U0126) and autophagy inhibitor (3MA). In brief, following experimental young ischemic stroke, the long-term promotion of the neurogenesis in the young gerbil’s hippocampal dentate gyrus by upregulating the phosphorylation of ERK signaling pathway and maintaining autophagy-related protein levels, it overtly improved the neurological function and cognitive and memory function.

## Introduction

Stroke is the second leading cause of death and the third leading cause of disability worldwide [1]. Ischemic stroke accounts for 87% of the total incidence of stroke, which is characterized by a sudden cessation of oxygen and blood supply due to arterial occlusion in local cerebral tissue [2, 3]. Recent studies found that more than 2 million young people worldwide suffer from ischemic stroke each year, accounting for 15–18% of all ischemic stroke [4, 5]. Most current investigations define the young ischemic-stroke population as being between the ages of 18 and 45 years [4]. According to recent epidemiological surveys, in contrast to the decreasing incidence of stroke in the elderly, the incidence of ischemic stroke in young people has been increasing yearly and trending upward as a percentage of the total stroke population [4, 6]. Ischemic stroke in youth has become a serious public health burden [7]. Stroke in youth not only affects the physical function and cognitive ability of adolescents, but also may cause lifelong intellectual disability, which imposes a heavy economic burden on society and families [8, 9].

It is well known that the proliferation and differentiation of endogenous neural stem cells (NSCs) occurred in the subventricular zone of the lateral ventricle, subventricular zone of the hippocampal dentate gyrus, cerebral cortex, and choroid plexus in the brain following transient cerebral ischemia/reperfusion [10–13]. Newly born neurons and glial cells migrate toward the site of injury to replace and repair damaged neural tissue cells, participating in the repair process of neural and cognitive functions [14]. For neurological recovery and reconstruction after cerebral ischemic injury, the main focus is on promoting the proliferation of endogenous NSCs and NSC transplantation. An increase of endogenous NSC proliferation and differentiation is an effective way to promote neurological recovery after cerebral ischemia/reperfusion [15, 16].

ERK signaling is a master regulator of cell behavior, life, and fate [17]. It promotes neurogenesis by regulating the activation of transcription factors and gene expression [18]. Previous study proved that inhibition of MAPK/ERK signaling could aggravate hippocampal neuronal apoptosis, decrease neurogenesis, and impair the offspring’s cognitive performances [19]. Conversely, increased phosphorylation of Erk1 and Erk2 in a mice model of scopolamine-induced memory deficits activates their downstream target cAMP response-element binding protein, subsequently leading to increased neurogenesis and improved learning and memory [20].

Autophagy is the major intracellular degradation system by which cytoplasmic materials are delivered to and degraded in the lysosome [21]. It can provide sufficient energy for the process of neurogenesis and decreased levels of autophagy may lead to a shortage of energy supply and impede the process of neurogenesis [22]. In previous studies, Atg9a levels and LC3-II/LC3-I ratios were elevated during neurogenesis in neural stem cells derived from the forebrain [23]. Moreover, neurogenesis was decreased in neural stem cells from Atg5^−/−^ mice, but supplementation with methylpyruvate (an analog for the citric acid cycle that restores ATP availability) rescued the phenotype, indicating that neural stem cells require autophagy as an energy source to differentiate into neurons [24].

Many studies have been already reported that ischemic stroke is a well-recognized disease of aging, however, it is unclear how the age-dependent vulnerability occurs and what are the underlying mechanisms. In our previous studies, we reported that young gerbils (only 1-month-old) are much more resistant to transient cerebral ischemia than the adult [25–28]. We also investigated changes of inflammatory cytokines, antioxidant redox system, and trophic factors in young gerbil following transient (5 mins) cerebral ischemia/reperfusion [29–31]. In our further study, we found that much aggravated neuronal death occurred in the CA1 region following 5 min of transient cerebral ischemia/reperfusion in 2-month-old gerbils compared with that in 1-month-old one. However, its recovery of neurological function and cognition and memory was mainly faster than those in the old gerbils. It is well known that recovery of neurological function and cognition and memory was closely related to neurogenesis in the dentate gyrus of hippocampus after transient cerebral ischemia [32–34]. Therefore, in the present study, we investigate the difference of neurogenesis and related proteins in the dentate gyrus of hippocampus after transient cerebral ischemia between young (2-month-old) and old (15-month-old) gerbils to explore the reason why the recovery of neurological function and congnition and memory was faster in young.

## Result

### Changes of neurological function and memory and learning abilities in young and old gerbils after ischemia/reperfusion (I/R)

**Fig. 1:**
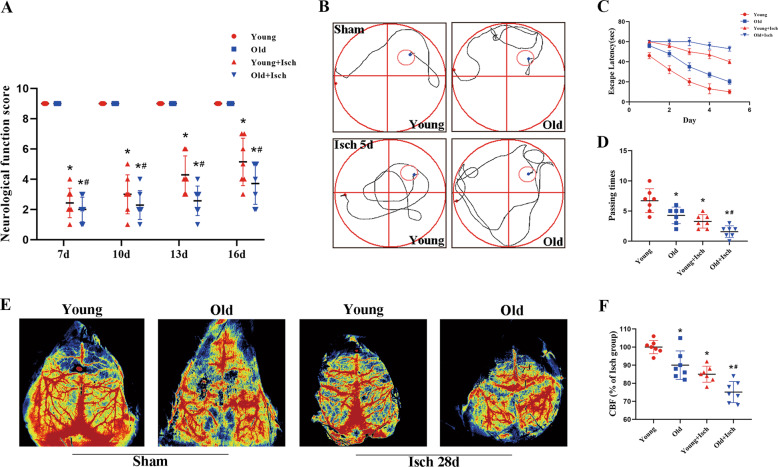
Observation of neurological function and recovery. Neurological-function score of young and old gerbils after I/R (**A**). The maps of computer printouts of the swimming trajectories on the fifth day of each group (**B**). The time spent in the removed escape-platform quadrant (**C**). Number of crossings over the original platform position by gerbils (**D**) (*n* = 7 per group; *p < 0.05, significantly different from the corresponding young group, #*p* < 0.05, significantly different from the young I/R group). Bars indicate mean ± SD.

In this study, we investigated the neurological function in gerbils after I/R. We scored the neurological function of young and old gerbils at 7, 10, 13 and 16 days after I/R. The results in Fig. 1A showed that there was no difference in neurological score between young and old gerbils before I/R. However, it was significantly decreased in both young and old gerbils after I/R. Following the time course of I/R, we found an increased tendency with neurological score in both young and old groups. However, we also found that the neurological score in all young gerbils were significantly higher than that in the corresponding groups of the old one after I/R. The results showed that the recovery of neurological function in the young gerbils was significantly faster than that in the old groups following I/R.

The Morris water maze results shown in Fig. 1B, D were used to investigate the changes on learning and memory deficits between young and old gerbils after I/R. The results showed that the time of escape latency in the I/R groups was significantly longer than that in the normal groups. In all normal groups, the time of escape latency in the young gerbils was shorter than that in the old one. In all I/R groups, compared with the old gerbils, the time of escape latency in the young gerbils was longer. The number of times the gerbils crossed the former location of the platform was decreased in both the old and young I/R groups compared with those in the normal groups. Compared with the old I/R group, the number of times the gerbils crossed the former location of the platform was increased in the young I/R group.

### Changes of cerebral blood flow in young and old gerbils after I/R

The results about cerebral blood flow shown in Fig. 1E, F were used to describe the differences about prognosis and ischemic extent between young and old gerbils after I/R. The results displayed that the cerebral blood flow in the I/R groups was distinctly less than that in the sham groups. We found, after I/R, that recovery of cerebral blood flow in young groups was higher than that in the old one.

### Changes of cell proliferation and neuroblast differentiation in the dentate gyrus (DG) of hippocampus in young and old gerbils after I/R

**Fig. 2:**
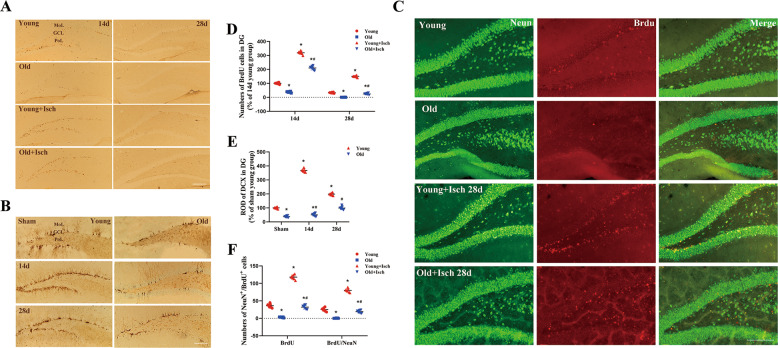
Observation of cell proliferation and neuroblast differentiation. Immunohistochemistry for BrdU in the DG region of the young and old of the sham and ischemia groups (**A**). Immunohistochemistry for DCX in the DG region of the young and old of the sham and ischemia groups (**B**). Double-immunofluorescence staining for BrdU, NeuN, and merged images in the DG 28 days after I/R in the young and old groups (**C**). The number of BrdU cells after I/R in the young and old groups. Data are represented by the mean number of BrdU cells per animal (**D**). Relative optical density as % of DCX immunoreactive structures in the young and old groups (**E**). Quantification of BrdU/NeuN cells at 28 days post ischemia. Data are represented by the mean percentage per animal (**F**). Scale bar = 100 μm (*n* = 7 per group; *p < 0.05, significantly different from the sham young group at the same reperfusion time point, #*p* < 0.05, significantly different from the corresponding young I/R group at the same reperfusion time point). Bars indicate mean ± SD.

In this study, we observed the long-term cell proliferation in the hippocampal DG of young and old gerbils used by 5-bromo-2′-deoxyuridine(BrdU) immunohistochemistry following I/R (Fig. 2A, D and Supplementary Fig. 2A). In all normal groups, the BrdU immunoreactive cells in the DG of young gerbils were more than those in the DG of old gerbils. In addition, in all I/R groups, we found, the BrdU immunoreactive cells in the DG of young gerbils were increased significantly at 14 days after I/R compared with those in the normal young gerbils and the old gerbils at 14 days after I/R. At 28 days after I/R, the number of BrdU immunoreactive cells in the young gerbils were decreased in the DG compared with those in the young gerbils at 14 days after I/R, but more than those in the corresponding old group.

We also observed differences of neuroblast differentiation in the DG of young and old gerbils following I/R by DCX immunohistochemistry (Fig. 2B, E and Supplementary Fig. 2B). In normal sham group, DCX immunoreactive neuroblasts were readily detected in the SGZ of the DG in the young gerbils. However, a few immunoreactive neuroblasts (30% of young group) were detected in the SGZ of the DG in the old group. In 14 days I/R group, the number of DCX immunoreactive neuroblasts increased obviously 3.5 fold in the SGZ of the DG in the young gerbils compared with that in the young sham group. Besides, the number of DCX immunoreactive neuroblasts in 14 days after I/R of the old group was obviously less than that in 14 days after I/R of the young group, while its level was slightly increased compared with that in the old sham group. In 28 days after the I/R group, the number of DCX immunoreactive neuroblasts decreased in the young group compared with 14 days after the I/R group, but it was still more than that in the young sham group. However, the number of DCX immunoreactive neuroblasts in 14 days after I/R was similar to that in the old sham group. Therefore, we found that immunoreactive cells of DCX were much increased in the young group than those in the old group before and after I/R. The dendritic protrusions of DCX cells were well developed in the young groups compared with the old groups.

We performed NeuN/BrdU double-immunofluorescence staining to elucidate the transformation of newly generated BrdU cells into mature neurons (Fig. 2C, F and Supplementary Fig. 2C). In the young sham group, we found a small number of BrdU cells colocalized with NeuN in the DG of hippocampus, whereas almost no BrdU and NeuN colocalized cells were present in the DG of hippocampus of the old sham group. In 28 days after I/R of young group, many BrdU/NeuN double immunoreactive cells were observed in the DG, some of them were observed in granule cell layers. The number of BrdU and NeuN colocalized cells in the young group was significantly more than in the old group.

### Differences of related protein-expression levels of Erk1/2 signaling pathway in the hippocampus after I/R between young and old gerbils

**Fig. 3:**
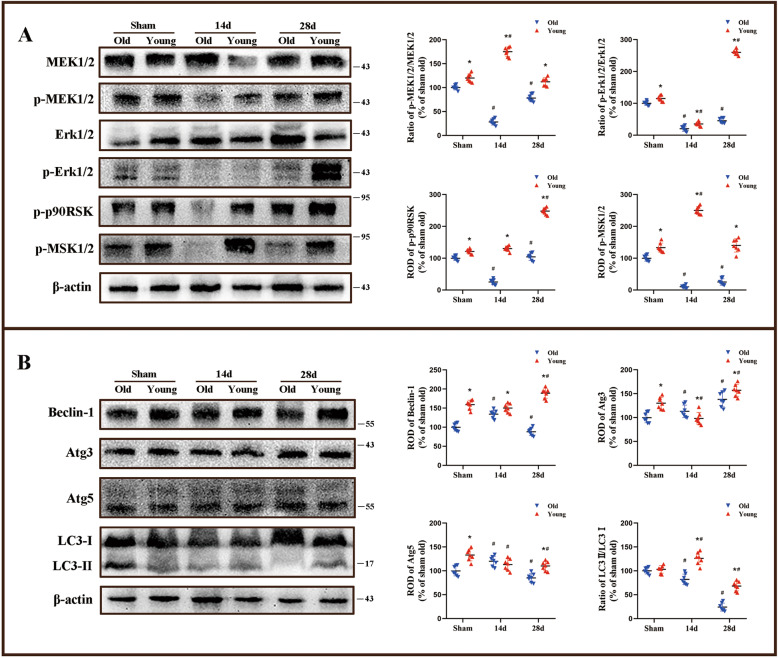
Observation of Erk1/2 signaling and autophagy-related protein levels. Western blot analysis of p-MEK1/2, MEK1/2, p-Erk1/2, Erk1/2, p-p90RSK and p-MSK1/2 in the hippocampus of the young and old groups after I/R (**A**). Western blot analysis of Beclin-1, LC3-I, LC3-II, Atg3, and Atg5 in the hippocampus of the young and old groups after I/R (**B**) (*n* = 7 per group; *p < 0.05, significantly different from the old group at the same reperfusion time point, #*p* < 0.05, significantly different from the sham group of the same age). Bars indicate mean ± SD.

Erk1/2 signaling pathway, including MEK1/2, Erk1/2, p90RSK, and p-MSK1/2, had been identified as a potentially important role in cerebral I/R injury. In our study, we found inhibition of the ERK1/2 pathway in the hippocampus of 14 and 28 days after I/R old gerbils, which was observed by decreased phosphorylation of MEK1/2, ERK1/2, p90RSK, and p-MSK1/2 (Fig. 3A and Supplementary Fig. 3A). However, the levels of phosphorylation of MEK1/2, ERK1/2, p90RSK and p-MSK1/2 were significantly increased in the hippocampus of 14 and 28 days after I/R young gerbils. These results indicated that Erk1/2 signaling pathway was significantly activated following I/R when the gerbils were young.

### Differences of expression levels of autophagy-related proteins in the hippocampus after I/R between young and old gerbils

Compared with the old groups, the overall levels of autophagy-related proteins, including Beclin-1, Atg3, -5, and LC3-II/-I in young groups were higher at the corresponding time points (Fig. 3B and Supplementary Fig. 3B). In detail, the level of Beclin-1 in the hippocampus increased significantly at 28 days after I/R in young gerbils compared with that in young sham group and the corresponding old I/R group. We found that the changes of tendency on the levels of Atg3 and Atg5 in the hippocampus were similar with young and old gerbils following I/R, however, its levels were slightly increased in all young groups compared with those in the corresponding time point of old groups. The overall level of LC3-II/-I in the hippocampus in young group was higher than that in the old group following all I/R groups, especially in 28 days after I/R groups. These results indicated that the expression levels of autophagy-related proteins in the hippocampus of young gerbils after I/R were higher than those in the old group.

### Levels of neurological-function recovery, memory and learning abilities, and cerebral blood flow following I/R in young gerbils were reversed by treatment of U0126 or 3MA

**Fig. 4:**
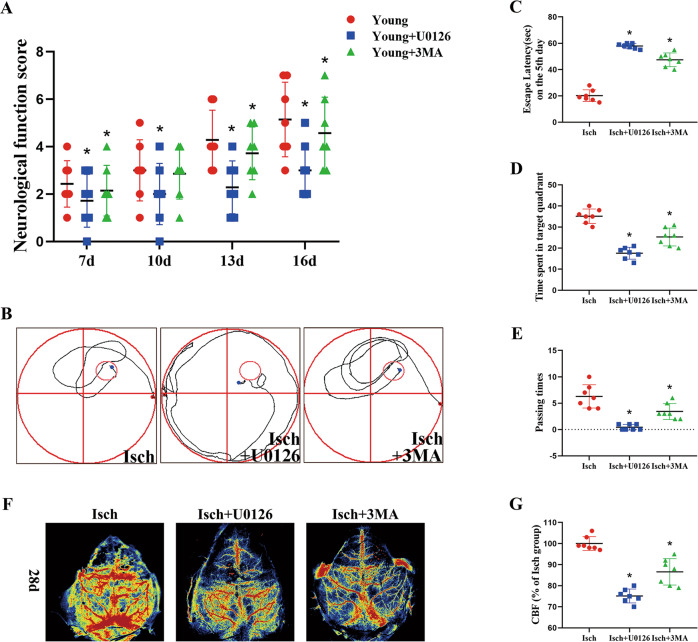
Change of neurological function and recovery by U0126 or 3MA treatment. Neurological-function score of young gerbils treated with U0126 or 3MA after I/R (**A**). The maps of computer printouts of the swimming trajectories on the fifth day of each group (**B**). The escape latency on the 5th day of each group (**C**). The time spent in target quadrant on the last day of each group (**D**). Number of crossings over the original platform position by gerbils (**E**) (*n* = 7 per group; *p < 0.05, significantly different from the young group at the same reperfusion time). Bars indicate mean ± SD.

In this study, we investigated the critical effects of Erk1/2 signaling and autophagy pathway on neurological function, memory and learning abilities, and neurogenesis after I/R in young gerbils used by U0126 of Erk1/2 inhibitor and 3MA of autophagy inhibitor. Compared with the young I/R group, the score of neurological function was decreased in the U0126- and 3MA-treated groups following 7, 10, 13, and 16 days after I/R (Fig. 4A). Our results tested by Morris water maze also showed that the abilities of memory and learning became worse induced by treatment with U0126 and 3MA following I/R, which were observed by prolonging the time of escape latency, decreasing the time of crossing platforms (Fig. 4B, E). In addition, the levels of the cerebral blood flow in the U0126- and 3MA-treated I/R groups were distinctly lower than those in the young I/R groups (Fig. 4F, G). These results showed that the neurological function recovery and memory and learning abilities in the young I/R gerbils were inhibited by treatment with U0126 and 3MA.

### Cell proliferation and neuroblast differentiation in the dentate gyrus (DG) induced by I/R in young gerbils were reversed by treatment of U0126 or 3MA

**Fig. 5:**
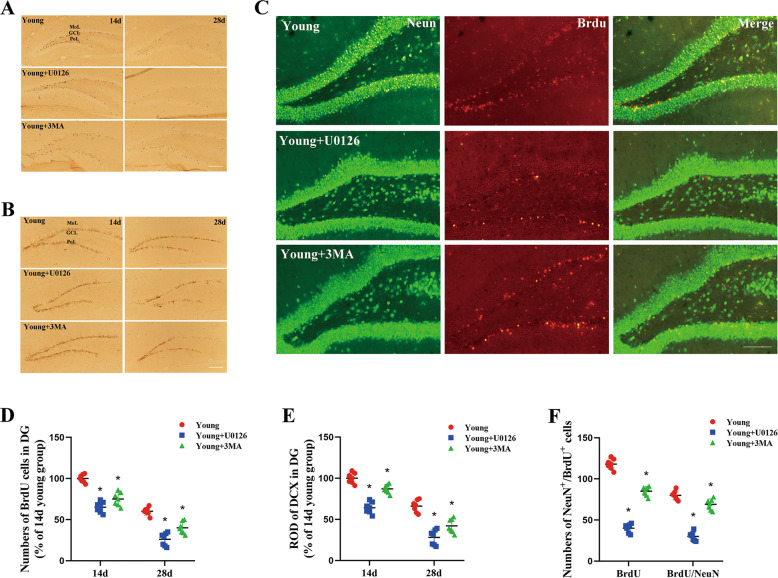
Change of Cell proliferation and neuroblast differentiation by U0126 or 3MA treatment. Immunohistochemistry for BrdU in the DG region of the young gerbils treated with U0126 or 3MA after I/R (**A**). Immunohistochemistry for DCX in the DG region of the young gerbils treated with U0126 or 3MA after I/R (**B**). Double-immunofluorescence staining for BrdU, NeuN, and merged images in the DG 28 days after I/R in the young gerbils treated with U0126 or 3MA (**C**). The number of BrdU cells after I/R in the young gerbils treated with U0126 or 3MA. Data are represented by the mean number of BrdU cells per animal (**D**). Relative optical density as % of DCX immunoreactive structures in the young gerbils treated with U0126 or 3MA (**E**). Quantification of BrdU/NeuN cells at 28 days post ischemia. Data are represented by the mean percentage per animal (**F**). Scale bar = 100 μm (*n* = 7 per group; *p < 0.05, significantly different from the young group at the same reperfusion time). Bars indicate mean ± SD.

Compared with the young I/R group, the cell proliferation by BrdU immunohistochemistry and neuroblast differentiation by DCX immunohistochemistry in the U0126- and 3MA-treated I/R groups were significantly reduced (Fig. 5 and Supplementary Fig. 5). In detail, we found that about 50% of BrdU immunoreactive cell, DCX immunoreactive neuroblasts and BrdU/NeuN colocalized cells significantly were decreased in the DG of hippocampus at 28 days after I/R in the U0126-treated I/R groups compared with those in the young I/R groups. In the 3MA-treated I/R groups, BrdU immunoreactive cells, DCX immunoreactive neuroblasts, and BrdU/NeuN colocalized cells were decreasd about 20–30% of the young I/R groups following 28 days after I/R. These results showed that the inhibition of Erk1/2 signaling pathway was pivotal for blocking the cell proliferation and neuroblast differentiation following young I/R groups.

### More activation of Erk1/2 signaling and autophagy pathway induced in young I/R was reversed by treatment with U0126 and 3MA, respectively

**Fig. 6:**
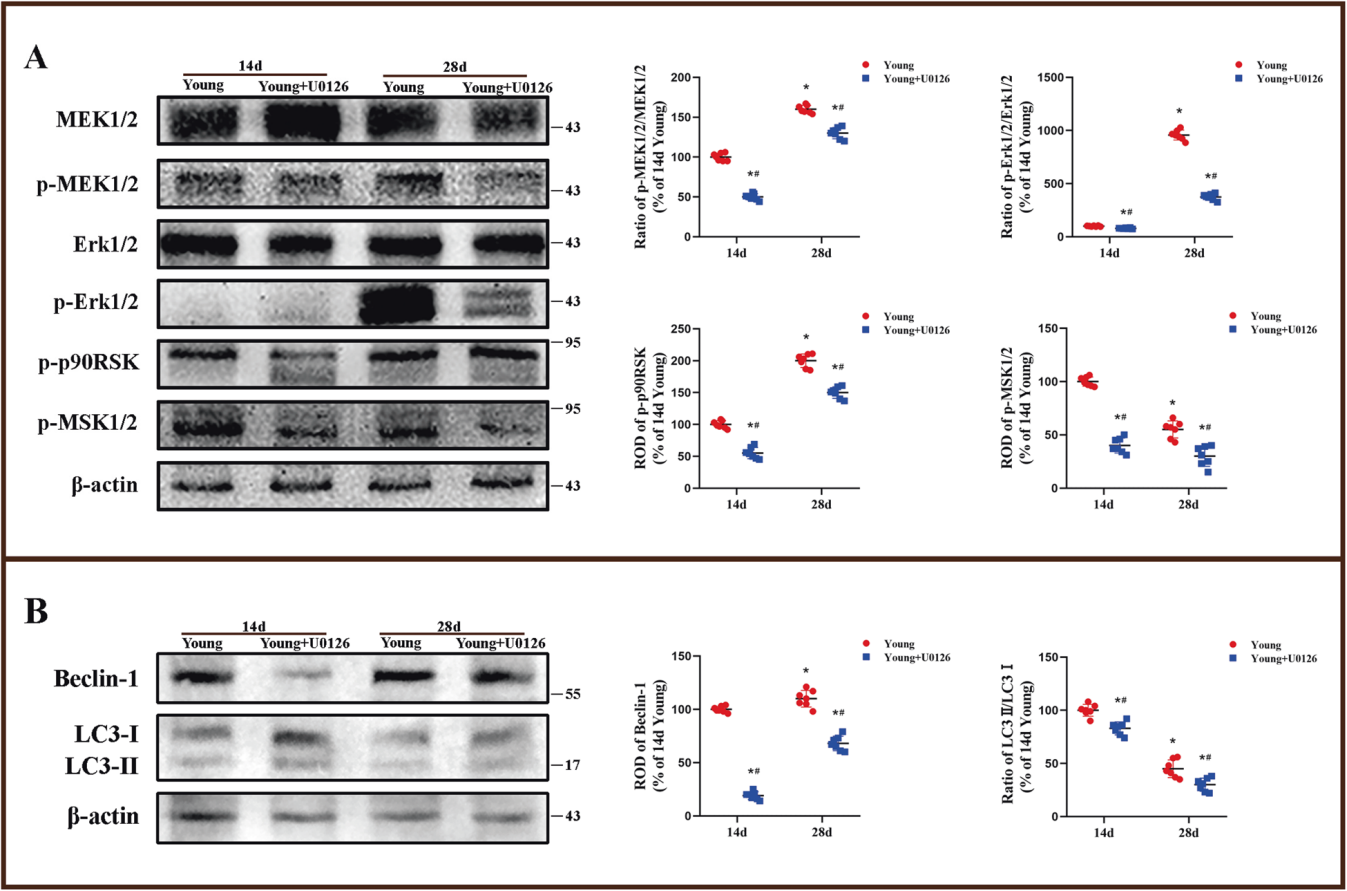
Change of Erk1/2 signaling and autophagy-related protein levels by U0126 treatment. Western blot analysis of p-MEK1/2, MEK1/2, p-Erk1/2, Erk1/2, p-p90RSK and p-MSK1/2 in the hippocampus of the young gerbils treated with U0126 (**A**). Western blot analysis of Beclin-1, LC3-I and LC3-II in the hippocampus of the young gerbils treated with U0126 (**B**) (*n* = 7 per group; *p < 0.05, significantly different from the 14 days young group, #*p* < 0.05, significantly different from the young group at the same reperfusion time). Bars indicate mean ± SD.

In this study, we found significantly that the expressions of p-MEK1/2/MEK1/2, p-Erk1/2/Erk1/2, p-p90RSK, and p-MSK1/2 in the hippocampus of U0126-treated group were suppressed compared with the young I/R group (Fig. 6A and Supplementary Fig. 6A). In addition, the expression levels of Beclin-1 and LC3-II/-I were also decreased in the hippocampus by U0126 treatment compared with those in the corresponding 14 and 28 days after I/R (Fig. 6B and Supplementary Fig. 6B).

**Fig. 7:**
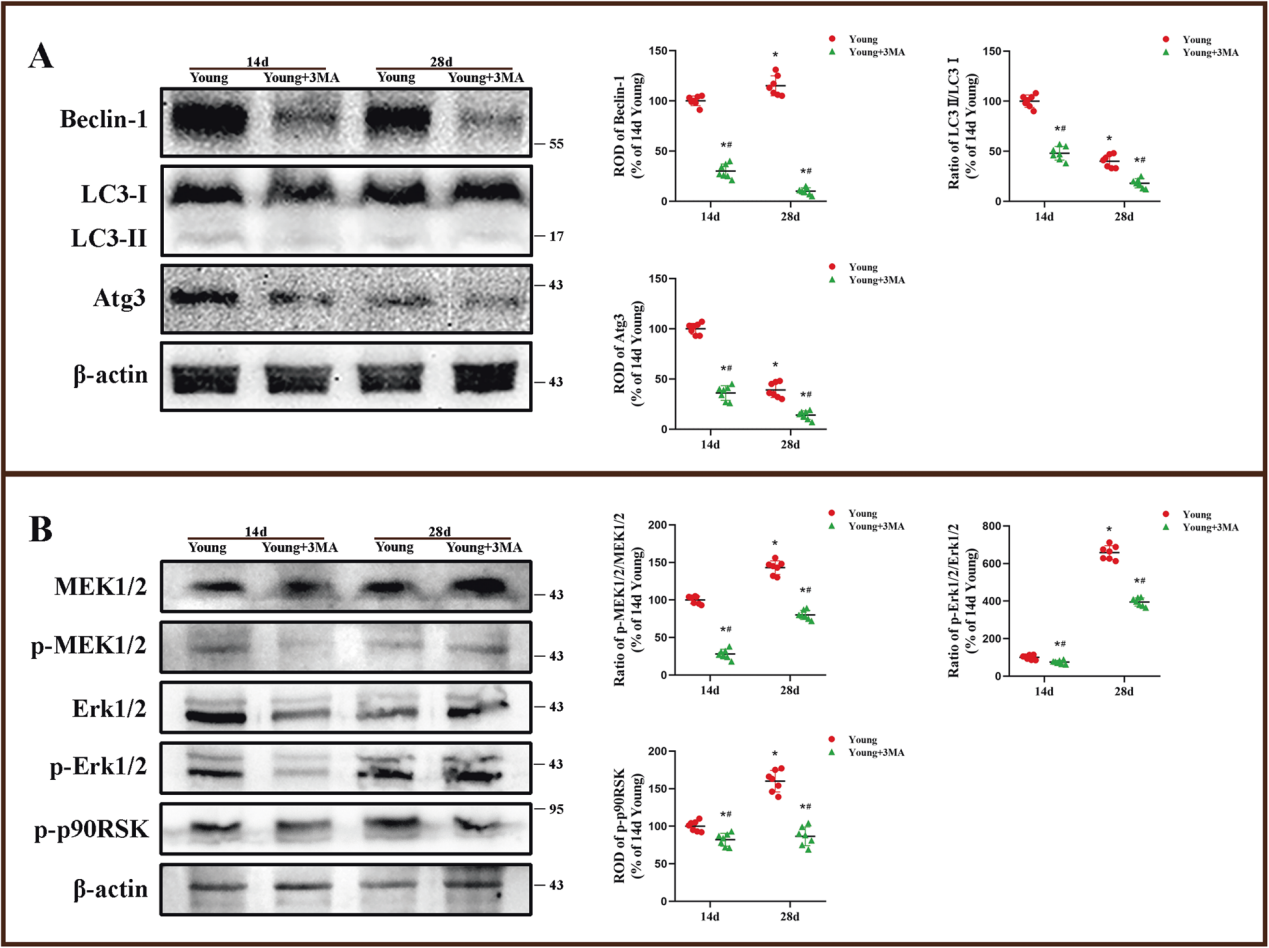
Change of Erk1/2 signaling and autophagy-related protein levels by 3MA treatment. Western blot analysis of Beclin-1, LC3-I, LC3-II, and Atg3 in the hippocampus of the young gerbils treated with 3MA (**A**). Western blot analysis of p-MEK1/2, MEK1/2, p-Erk1/2, Erk1/2, and p-p90RSK in the hippocampus of the young gerbils treated with 3MA (**B**) (*n* = 7 per group; *p < 0.05, significantly different from the 14-d young group, #*p* < 0.05, significantly different from the young group at the same reperfusion time). Bars indicate mean ± SD.

Compared with the corresponding young I/R group, we found significantly that the expressions of Beclin-1, Atg3 and LC3II/I in the hippocampus were inhibited by 3MA treatment in 14 and 28 days after I/R groups (Fig. 7A and Supplementary Fig. 7A). In addition, the levels of p-MEK1/2, p-Erk1/2, and p-p90RSK, as the essential proteins of Erk1/2 signaling, were also inhibited by 3MA treatment in the hippocampus of the young following 14 and 28 days after I/R (Fig. 7B and Supplementary Fig. 7B).

## Discussion

It is well known that neurogenesis occurs in the neurogenic niches of the postnatal brain, such as hippocampal DG, following transient forebrain ischemic stroke in rodents including mice, gerbils, and rats [35, 36]. Neurogenesis in the hippocampal DG region declines with age in normal aging process [37, 38]. However, we found that few studies focus on the changes of neurogenesis in young gerbils when suffered from transient forebrain ischemic injury. In this study, our results first showed that, after forebrain cerebral ischemia, long term of more increased neurogenesis observed by immunohistochemistry of BrdU and DCX in the hippocampal DG occurred in the young gerbils than the old gerbils.

The hippocampal DG region is an important region for maintenance of neuroplasticity, memory spatial learning capacity, and neurorehabilitation capacity [39, 40]. Age-dependent rodents that typically exhibit cognitive decline were associated with a reduced hippocampal volume, altered neuroplasticity, neurogenesis, and synaptic plasticity [41–43]. Our present study showed that neurological function and cognitive capability in young gerbils were much better than old gerbils though suffering from the equal ischemic injury. This study well explained that the better recovery of cognitive function in the young stroke patients was shown by previous studies [44, 45].

Hippocampal neurogenesis has been shown to be an important event in cognitive and neurological recovery [32, 46, 47]. Its increased neurogenesis and neuroplasticity significantly improve memory spatial learning capacity and neurorehabilitation capacity [48, 49]. Cerebral injury caused by transient forebrain ischemic stroke leads to cognitive dysfunctions, and this process is accompanied by elevated neurogenesis in the neurogenic niches of the postnatal brain [50, 51]. Many studies have already been suggested that the neurogenesis, including cell proliferation and differentiation of neuroblasts into mature neurons by some stimuli, is the key role to promote neurological rehabilitation and cognitive dysfunction in some common neurodegenerative diseases, including stroke in rodents [52, 53]. In the present study, our results demonstrated that the capability of neurogenesis in young gerbils after long-term I/R injury is directly proportional to the recovery of neurological function and cognitive ability, on the contrary, this capability of old gerbils is obviously insufficient. Some previous studies also proved that Zeb2-/Axin2-enriched BMSC-derived exosomes stimulated endogenous neurogenesis, which induced functional recovery after stroke [54]; Ginseng total saponins can improve neurological deficits after focal cerebral ischemia by inducing endogenous neural stem cell activation [55]. Ligusticum chuanxiong had the ability to protect neurons by promoting the endogenous proliferation of neuroblasts and production of neural differentiation factor in rats after ischemia injury [56]. Also, damage to the hippocampus caused by head trauma, ischemia, stroke, status epilepticus, and Alzheimer’s disease restrained neurogenesis and thereby prevented the recovery of neurological function [57]. Diets high in fat and refined sugars contributed to cognitive decline and dementia by reducing hippocampal neurogenesis and impairing spatial memory [58]. Therefore, our study indicated that the increase of neurogenesis in young gerbils after I/R injury was closely related to the recovery of neurological function and actively affected cognitive ability.

It is well known that neurogenesis largely depends on molecular and genetic inputs, such as growth factors and cellular signaling pathways, creating a microenvironment, or niche, for neural stem/precursor cells [59–61]. Moreover, these processes are also modulated during many pathological states, such as ischemia [62, 63]. ERK/MAPK is a signaling pathway essential for cell growth and differentiation, which facilitates cell cycle exit and differentiation into neurons by regulating the proliferation and differentiation of neural stem cells [64]. Previous studies have shown that activation of the Erk signaling pathway significantly enhances cerebral ischemia-induced neurogenesis and promotes the neuroblast migration into mature neurons [65]. Our results showed that Erk1/2 signaling in the hippocampus of young gerbils was more activated after transient cerebral ischemia, which may lead to neurogenesis enhanced in the DG. Our further results showed that blocking the transduction of ERK signaling pathway by applying U0126 significantly reduced the level of neuroblast differentiation and prevented newborn cells’ migration into neurons in DG region, and eliminated the rehabilitation advantage of neurological function and spatial memory function in young gerbils. These results consisting with previous studies have shown that activation of ERK1/2 improves learning memory by participating in synaptic-plasticity formation and promoting proliferation of granule cells in DG [66]. Some previous studies also proved that the novel exercise-induced hormone irisin protected against neuronal injury via activation of ERK1/2 signaling pathways and contributed to the neuroprotection of physical exercise in cerebral ischemia [67]. The neuroprotective effects of Tongxinluo on focal cerebral ischemia and reperfusion injury in rats associated with the activation of the MEK1/2/ERK1/2/p90RSK signaling pathway [68]. On the contrary, methylcobalamin activated the ERK1/2 pathway, whereas ERK1/2 inhibitors diminished its effects in the in vitro and in vivo models and decreased the neuroprotective effects in cerebral ischemia/reperfusion injury [69]. U0126, which inhibits Erk1/2 phosphorylation, enhanced ischemia-induced cell death [70]. Taken together, our results suggest improved neurological- and memory-function recovery in young gerbils after ischemia/reperfusion by promoting cell proliferation and differentiation and increasing or activating the expression level of Raf/MEK/ERK signaling pathway.

Autophagy is a ubiquitous cytoprotective process that plays a critical role in the degradation and recycling of cellular components, including damaged organelles, pathological proteins, and malfunctioning macromolecules, to maintain cellular and tissue homeostasis in vivo [71]. Autophagy regulates several physiological and pathological processes through lysosomal-degradation processes, such as myelin degradation, myelin development, and nerve regeneration [72]. Autophagy promotes the proliferation and differentiation of neural stem cells by regulating reactive oxygen species and reducing intracellular oxidative-stress levels [73–75]. Our results showed that higher autophagy levels were maintained in the young gerbils after I/R. Recent studies have shown that nanoscale electrical stimulation enhances autophagic signaling, promotes the differentiation of NSCs to mature neurons, and prevents neurodegeneration [74]. Therefore, our present study showed that promoted cell proliferation, neuroblast differentiation, and neural regeneration in the DG region after I/R were concerned to maintaining autophagy levels. Beclin-1/ATG/LC3-II-dependent pathway is regarded as canonical autophagy [76, 77]. Our further results showed that 3MA, as an autophagy inhibitor, significantly inhibited cell proliferation and neurogenesis after I/R and attenuated the recovery of neurological and spatial memory function in young gerbils. In the previous studies, deficiency or inhibition of Beclin-1 or ATG family members significantly decreased LC3-II and prevented the autophagy production in vivo and invitro cerebral ischemic stroke [78, 79]. Inhibition of autophagy by knockdown of Atg5 or other autophagy-related genes such as FIP200 impaired neuronal differentiation of DG and SGZ neural stem cells by reducing the number of neonatal neurons and neuronal maturation [80, 81]. Therefore, our results in the present study demonstrated that maintenance or elevated autophagy level in the young gerbils hippocampus stimulated neurogenesis, which promoted cell proliferation, neuroblast differentiation, and neural regeneration in the DG region after I/R.

Some previous studies proved that in rats with spinal cord injury, physical exercise activated the ERK1/2 signaling pathway, leading to neurological rehabilitation and improved motor function [82]; Electroacupuncture significantly increased the phosphorylation of ERK, thus improving the neurological-function recovery in cerebral ischemia/reperfusion injury [83]. U0126, the MEK1/2 inhibitor, blocked exercise-induced phosphorylation of ERK1/2, thereby affecting neurological-rehabilitation [84]. Inhibition of ERK1/2 by U0126 reversed neurological function recovery in exercised ischemic rats [85]. Activation of the ERK signaling pathway significantly enhanced cerebral ischemia-induced neurogenesis and promoted the migration of newborn cells [65]. Inhibition of MAPK/ERK signaling pathway could block hippocampal neurogenesis in newborn rats [19]. In the previous studies, overexpression of enhanced autophagy in spinal cord injury improved neurological-function recovery [86]. In spinal cord injury model rats, autophagy has been shown to reduce neuronal injury and promote neurological recovery by inhibiting neuronal apoptosis, whereas inhibition of autophagy by 3MA reversed this phenomenon [87]. Autophagy played an extremely important role in the regulation of stem cells and was essential for neurodevelopment and embryonic neurogenesis [88]. Autophagy-related genes were of great importance in adult neurogenesis [89]. Impaired autophagy was associated with a decline in adult neurogenesis, which could be reversed by autophagy activation [90]. Recent studies have shown that transcranial direct current stimulation can play a therapeutic role in inducing neurological rehabilitation through neurogenesis regulated by Notch1 signaling after cerebral ischemia/reperfusion [91]. Forced-limb use could enhance neurogenesis and neurological-function recovery after stroke in elderly rats [92]. Constraint-induced movement therapy enhanced neurogenesis and neurological rehabilitation after cerebral ischemia/reperfusion [93]. Treadmill exercise promoted neurogenesis through upregulation of Wnt/β-catenin signaling pathway, and ameliorated neurological deficits caused by cerebral ischemia/reperfusion, whereas inhibitors eliminated exercise-promoted neurogenesis in the ischemic penumbra [94].

In conclusion, enhanced Erk1/2 or autophagy signaling can promote neurogenesis and thus neurological rehabilitation. Based on the above analysis, our current study focused on confirming the positive correlation between neurological rehabilitation ability and neurogenesis ability after ischemic stroke in childhood, and confirmed that ERK and autophagy-signaling pathway were the key factors through positive and negative aspects, providing theoretical basis for rehabilitation treatment of childhood stroke. These results of our study suggest that more growth of cell proliferation, neuroblast differentiation and neural regeneration in the DG region of young gerbils after I/R promotes neurological recovery after I/R, which is mainly accomplished by upregulating the ERK signaling pathway and maintaining the level of autophagy.

## Materials and methods

### Experimental animals

Healthy male Mongolian gerbils (Meriones unguiculatus) were progeny of Mongolian gerbils freely obtained from the Experimental Animal Center, Kangwon National University, Chunchon, South Korea. All experimental animals are kept in the Comparative Medicine Center of Yangzhou University. Those animals were put into the experiment after one week of domestication. They were raised under the environment of adequate temperature (23 °C), humidity (60%) control, and a 12-h light/12-h dark cycle with free access to food and water. The National Institutes of Health Guide for the Care and Use of Laboratory Animals must be followed throughout the whole experimental procedures. All experimental investigation procedures were authorized by the Yangzhou University Institutional Animal Care and Use Committee (YIACUC-14-0015).

### Induction of transient cerebral ischemia

The forebrain ischemic stroke model was prepared based on the bilateral common carotid artery clamping method. After the gerbils were anesthetized by inhalation with isoflurane gas, the bilateral common carotid arteries were exposed along the midline of the neck and clamped simultaneously using arterial clamps. The blood flow of the central retinal artery was observed under fundoscopy. After complete interruption of blood flow and occlusion for 5 min, the bilateral arterial clamps were removed simultaneously. The fundoscope was used again to observe whether the blood flow in the central retinal artery returned to normal. In the sham-operated group, only both common carotid arteries were separated without arterial clamping. An automatic heating pad connected to a rectal thermometer was used intraoperatively to ensure that the body temperature of the experimental animals was maintained at 37 ± 0.5 °C. Postoperatively, they were placed in an incubator to maintain body temperature.

### Experimental grouping and drug administration

The gerbils were divided by age into a young group (2-months old, 30–35 g) and an old-age group (15-months old, 100–115 g). Subsequently, the young group continued to be randomly divided into Sham operation (sham group), Ischemic operation (Isch group), Ischemic operation with U0126 treatment (Isch + U0126 group), and Ischemic operation with 3MA treatment (Isch + 3MA group), and the elderly group was randomly divided into Sham operation (sham group) and Ischemic operation (Isch group). The surgical group was treated by ischemia for 5 min and reperfusion for 14 d or 28 d. There were 21 animals in each group.

BrdU treatment: To check the cumulative markers of newly generated cells in the dentate gyrus (DG) of the hippocampus after ischemia, the animals were administered intraperitoneal BrdU injections (50 mg/kg) twice daily from 1d, 2d, 4d, 6d, 8d, and 14 days after ischemia, respectively.

U0126 treatment: U0126 (0.5 mg/kg) was administered intraperitoneally to animals in the inhibitor group at 5 and 10 d postoperatively, respectively.

3MA treatment: 3MA (15 mg/kg) was administered intraperitoneally to the inhibitor group at 5 and 10 d postoperatively, respectively.

### Neurological function score

Neurological-function score was scored on an 9-point scale [95]. The evaluation was divided into three independent tests scored from 0 to 3 to determine the following parameters: [1] balance-beam test, place the gerbil on a 2.0-cm-diameter balance beam, if it can maintain balance for more than 30 s, then score 3; [2] grasp-traction test, prepare a 1.0-cm-diameter suspended nylon rope and place the gerbil on it, if the residence time on the rope exceeds 5 s, then score 3; [3] grid test, place the gerbil on a grid plate with an area after the grid test, the gerbil was placed on a grid plate with an area of 20 cm×40 cm, and the plate was quickly turned from horizontal to vertical, and if the gerbil stayed on the grid for more than 15 s, the score was recorded as 3. The sum of the scores of the three tests was taken as the neurological function score of the gerbils.

### Morris water maze experiment

The Morris water maze is a convenient and popular test to evaluate neurological rehabilitation and cognitive function [96]. Fill the apparatus with water (25 ± 1 °C) and familiarize the animals with the experimental environment prior to the start of the experiment. On the first day, all gerbils were allowed to swim freely. From day 2 to day 7, gerbils (*n* = 7) were trained to find the platform for 60 s and then rested on the platform for 15 s. The platform was visible above the water level at the end of the training. From day 8 to day 12, platforms were hidden 1.5 cm below the water surface and each gerbil was given 4 trials per day for a maximum of 60 s per trail. The waiting time to climb onto the hidden platform was recorded to assess learning ability. On day 13, the animals were subjected to a probing test. The hidden platform was removed and the number of times the gerbils crossed the previous location of the hidden platform was recorded to test memory function.

### Tissue processing for histology

All experimental animals were anesthetized with 2% pentobarbital sodium and then perfused with heart before execution. Briefly, 0.1 M phosphate buffer salt water (PBS) was infused to remove blood from the tissues, followed by 4% paraformaldehyde to fix the tissues. After fixation, the animal was decapitated and the brain tissues were placed in a 30% sucrose solution for approximately 24 h. Finally, the tissues were cut into 30-μm sections using a frozen sectioning machine for storage.

### Immunohistochemistry

With reference to previously published procedure, ABC–DAB immunohistochemistry was performed [69]. Each brain slice was washed three times with PBS and placed in 0.3% hydrogen peroxide (H_2_O_2_) for 20 minutes to eliminate endogenous peroxidase activity. This was followed by treatment with 5% normal goat serum for 30 minutes. The sections were incubated with diluted goat anti-DCX (1:100, Santa Cruz Biotechnology, cat. sc-8066), and mouse anti-BrdU (1:100, Santa Cruz Biotechnology, cat. sc-32323) at room temperature for 12 h. Subsequently, they were treated with the corresponding secondary antibodies, which were treated by diaminobenzidine color development. A negative-control test was performed with immune serum instead of primary antibody, which showed no immunoreactivity in all structures, to establish the specificity of the immunostaining. Tissue patches, dehydration, and coverslips were then performed. After the slides were allowed to dry completely, the staining results were observed under a microscope and photographed.

### Western blot analysis

Western blot experiment was carried out by referring to previously published documents [69]. Gerbils were anesthetized and executed, and brain tissues from the hippocampal region were taken and sorted out. Subsequently, the isolated brain tissues were pretreated for protein extraction. When ultrasonic tissues were homogenized, protein tissues were centrifuged, supernatants were aspirated and boiled. The protein concentration of each group was then determined using the BCA method. The extracted proteins were subjected to electrophoretic separation of the gradients and transferred to PVDF membranes. To prevent binding of nonspecific protein-binding sites to antibodies, the membranes were treated in a blocking solution containing 5% BSA for 90 min. Subsequently, rabbit anti-p-MEK1/2 (1:1000, Cell Signaling, cat.#9154), MEK1/2 (1:1000, Cell Signaling, cat.#8727), p-Erk1/2 (1:2000, Cell Signaling, cat.#4370), Erk1/2 (1:1000, Cell Signaling, cat.#4695), p-p90RSK (1:1000, Cell Signaling, cat.#11989), p-MSK1/2 (1:1000, Cell Signaling, cat.#9595), Beclin-1 (1:1000, Cell Signaling, cat.#3495), LC3A/B (1:1000, Cell Signaling, cat.#12741), Atg3 (1:1000, Cell Signaling, cat.#3415), Atg5 (1:1000, Cell Signaling, cat.#12994), and β-actin (1:1000, Cell Signaling, cat.#4970) were added overnight at 4 °C. Secondary antibodies were added and incubated at 37 °C for 1 h. Subsequently, the strips were placed in a Bio-Rad automated gel imaging system and exposed to imaging with a drop of MinECL chemiluminescent reagent on the surface for photography. The strips were analyzed for grayscale values using Image Pro Plus 6.0 to evaluate the relative expression levels of the proteins.

### Double immunofluorescence staining for BrdU and NeuN

DNA was first denatured by incubating the sections in 50% formamide/2 × standard saline citrate at 65 °C for 2 h and then in 2 N HCl at 37 °C for 30 min. The sections were sequentially treated with 0.3% hydrogen peroxide (H_2_O_2_) in PBS for 30 min and incubated with the mixture of mouse anti-BrdU (diluted 1:150, Roche, Germany) and rabbit anti-neuronal nuclei (NeuN, diluted 1:400, Millipore, Billerica, MA, USA) overnight at 4 °C after the denaturation process of DNA for BrdU detection. After washing 3 times for 10 min with PBS, the sections were incubated in a mixture of diluted Alexa Fluor 488 donkey anti-mouse (diluted 1:250, Invitrogen, Carlsbad, CA, USA) and Alexa Fluor 546 donkey anti-rabbit (1:250, Invitrogen, Carlsbad, CA, USA) for 2 h at room temperature. The immunoreactions were observed under a microscope (Axioscope, Carl Zeiss, Germany) and a laser-scanning microscope (LSM 710, Carl Zeiss, Germany).

### Statistical analysis

All experimental data were statistically processed using SPSS 27.0 software, and all data were expressed as X ± S. One-way ANOVA or two way ANOVA was used for the comparison of measures obeying normal distribution, and SNK and LSD methods were used for two-way comparison between groups. *p* < 0.05 was considered statistically significant.

## Supplementary information

Supplemental Material

## Data Availability

All data generated or analyzed during this study are available from the corresponding author on reasonable request.
