# Supplementary figures and images for: Targeting the Erk1/2 and autophagy signaling easily improved the neurobalst differentiation and cognitive function after young transient forebrain ischemia compared to old gerbils
Source: Cell Death Discov. 2022 Feb 26;8:87. doi: 10.1038/s41420-022-00888-8 (PMC8882190; doi:10.1038/s41420-022-00888-8)

Original data: Immunostaining

Figure.2

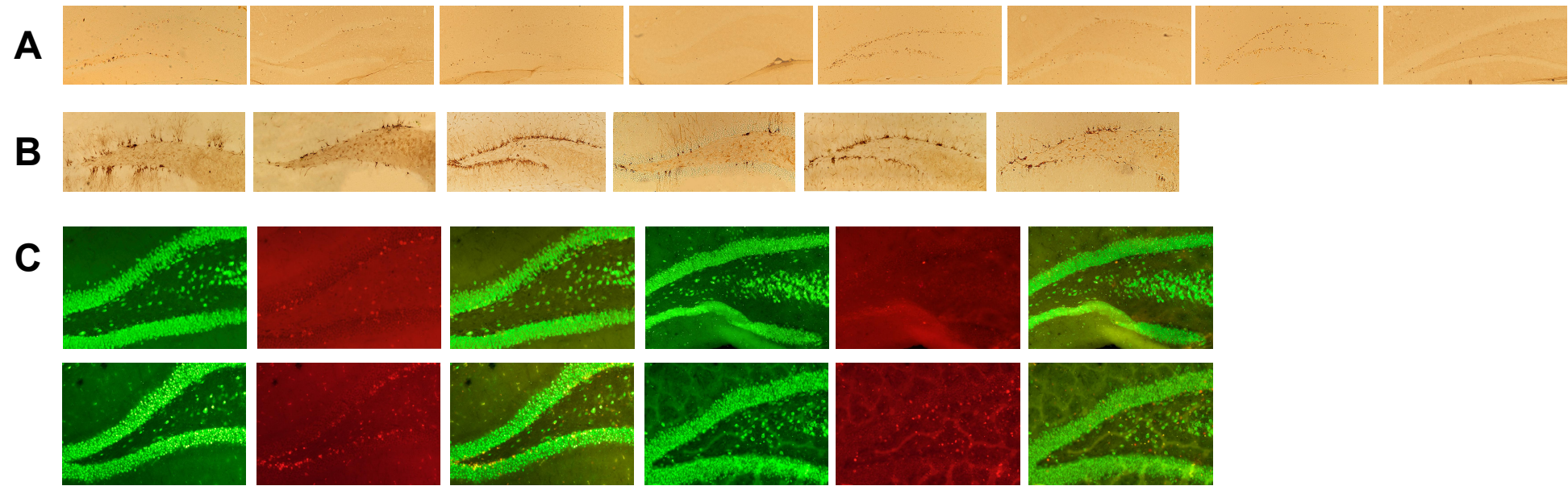

Figure.5

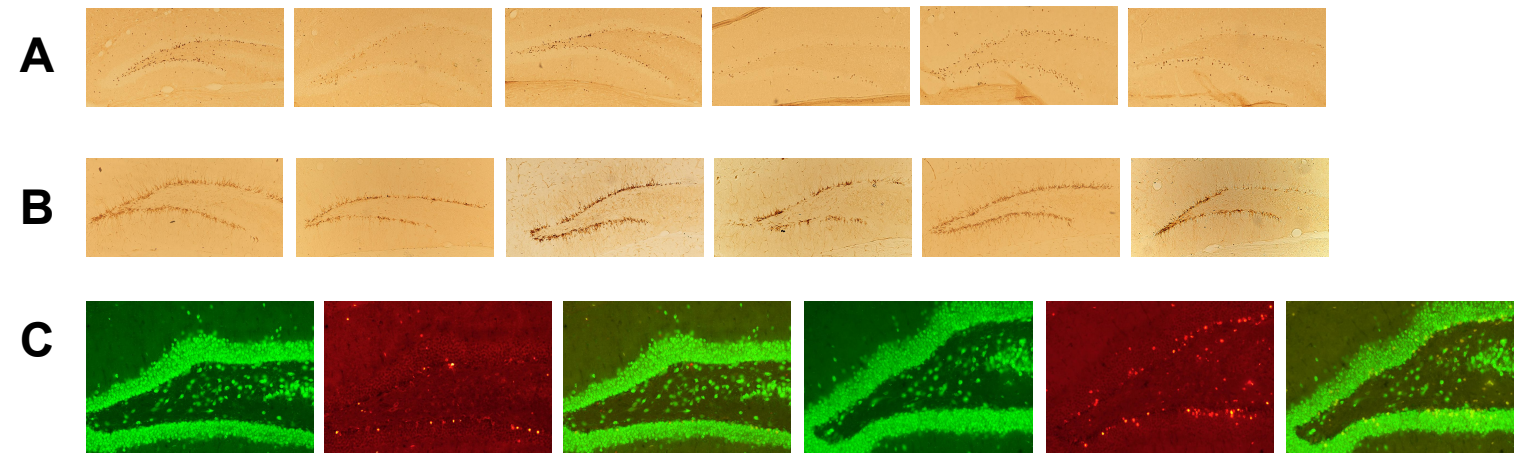

Original data: Western blot

Figure.3

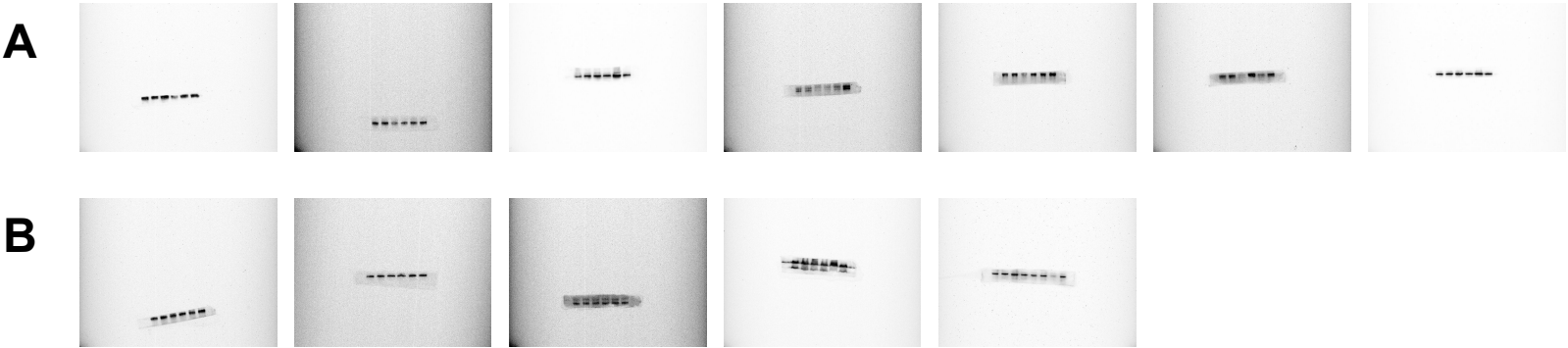

Figure.6

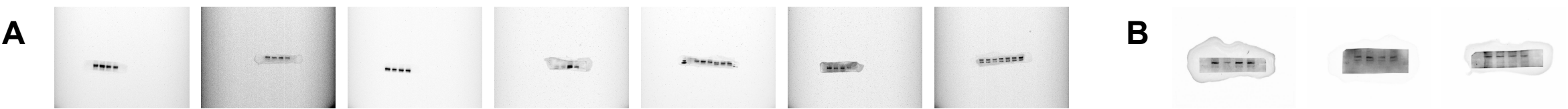

Figure.7

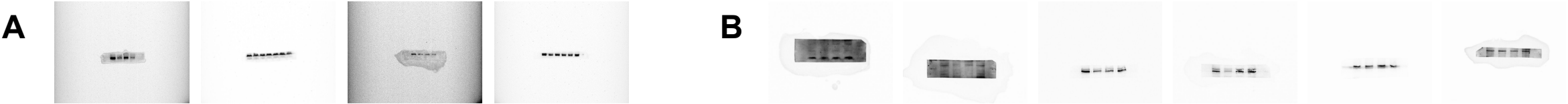

Supplement: Supplementary file 1 — Supplemental Material [file 41420_2022_888_MOESM1_ESM.pdf]
